# The association between residual cholesterol to high-density lipoprotein cholesterol ratio and type 2 diabetes risk in elderly populations

**DOI:** 10.3389/fendo.2025.1651416

**Published:** 2025-11-18

**Authors:** Jinting Zhang, Zhaoxiang Wang, Qianqian Wang, Yang Liu, Qi Shao, Ying Pan, Shao Zhong

**Affiliations:** 1 Department of Endocrinology, Kunshan Hospital Affiliated to Jiangsu University, Kunshan, Jiangsu, China; 2 Department of Critical Care Medicine, Shanghai East Hospital, Tongji University School of Medicine, Tongji University, Shanghai, China; 3 Department of General Medicine, Kunshan Hospital Affiliated to Jiangsu University, Kunshan, Jiangsu, China; 4 Biomedical Big Data Innovation Application Laboratory, The First People's Hospital of Kunshan, Kunshan, Jiangsu, China

**Keywords:** RC/HDL-C, T2D, cohort study, elderly population, incident T2D

## Abstract

**Purpose:**

The remnant cholesterol to high-density lipoprotein cholesterol ratio (RC/HDL-C) is a novel biomarker of metabolic disturbances. Prior studies have indicated a close association between elevated RC/HDL-C and the occurrence and progression of cardiovascular, cerebrovascular, and metabolic diseases. However, longitudinal data on the relationship between RC/HDL-C and type 2 diabetes (T2D) risk remain limited, particularly among high-risk elderly populations. This study aims to clarify the association between RC/HDL-C and incident T2D in Chinese adults, offering evidence for early prevention and detection of T2D.

**Patients and methods:**

A follow-up study was conducted in Kunshan, China, from January 2018 to July 2021, involving 7655 participants aged 60 years or older. Cox proportional hazards models were used to evaluate the independent effect of the RC/HDL-C level on the risk of T2D. The relationship between RC/HDL-C and T2D risk was visually demonstrated using the Kaplan-Meier method and restricted cubic splines (RCS).

**Results:**

During a median follow-up of 3.87 years, 783 participants (10.23%) developed T2D. A fully adjusted Cox proportional hazards model showed a positive and independent association between RC/HDL-C and T2D risk (HR = 1.12, 95% CI: 1.06-1.17, *P* < 0.001). The highest RC/HDL-C quartile (Q4) had the highest cumulative incidence of T2D (Log-rank test, *P* < 0.001). Furthermore, RCS analysis revealed a non-linear relationship between RC/HDL-C and T2D risk. Receiver operating characteristic (ROC) curve analysis revealed that the RC/HDL-C ratio exhibited the largest area under the curve (AUC = 0.601, 95% CI: 0.580–0.623), indicating modest but statistically significant predictive ability. Subgroup analysis further validated the robustness of these results.

**Conclusion:**

We found that older adults with elevated RC/HDL-C levels have a higher risk of incident T2D. RC/HDL-C is an independent predictor of incident T2D in the elderly and may serve as a valuable adjunct to enhance risk stratification within existing prediction models.

## 1 Introduction

With global socioeconomic development, lifestyle changes, and rising obesity, the incidence of type 2 diabetes (T2D) is increasing in parallel worldwide (1). The disease arises from multifactorial mechanisms driven by complex interactions among genetic, metabolic, and lifestyle factors. Obesity-related anthropometric measures remain simple and effective tools for screening dysglycemia, complemented by hypertension and physical activity levels (2–4). Moreover, lipid parameters, notably high-density lipoprotein cholesterol (HDL-C) and low-density lipoprotein cholesterol (LDL-C), continue to constitute foundational components for metabolic risk assessment and have been incorporated into diabetes risk prediction models (5). Abnormal glucose metabolism—including prediabetes and overt diabetes—is now a major public health concern. Notably, projections from 2017 estimate that more than 600 million individuals will develop prediabetes by 2045, with a similar number progressing to T2D (6). The prevalence of T2D is increasing most rapidly among the elderly population across all age groups. In China, studies indicate that elderly T2D patients face disproportionately higher complication rates, imposing significant clinical, familial, and societal burdens. Early diagnosis and intervention for T2D significantly improve cost-effectiveness and health outcomes (7, 8). Consequently, there is an urgent need for novel, easily measurable biomarkers to facilitate early screening and improve management of at-risk populations.

Previous research has confirmed that dyslipidemia is a risk factor for both macrovascular diseases and T2D. The limited effectiveness of statins in adequately managing diabetic dyslipidemia has shifted research interest from conventional lipid profiles to triglyceride-rich lipoproteins (TRLs) (9). Remnant cholesterol (RC), a novel non-traditional lipid parameter introduced recently, reflects cholesterol within TRLs that may more directly promote insulin resistance (IR) than triglycerides (TG), including remnants from very low-density lipoprotein (VLDL), intermediate-density lipoprotein (IDL), and chylomicrons (10). A large-scale Chinese cohort study showed that RC is more strongly associated with diabetes than LDL-C, possibly mediated through IR and proinflammatory pathways (11). HDL-C has antioxidant properties and an enhanced capacity for cholesterol efflux, whereas its relationship with T2D involves complex interactions among lipid metabolism, inflammation, and oxidative stress. Clinical data indicate an inverse relationship between HDL-C levels and the risk of T2D (12, 13). Reduced HDL-C levels are a key factor in the pathogenesis and etiology of T2D and prediabetes, and are consistently associated with increased atherosclerosis risk in both T2D patients and experimental models (14–16). Emerging evidence indicates that lipid or lipoprotein ratios have greater predictive value than individual lipid measures for T2D risk, as they more effectively capture interactions among lipid components (17–22). Although a high RC/HDL-C ratio is strongly linked to cardiovascular, cerebrovascular, and metabolic disorders, studies investigating its association with incident diabetes risk are limited, especially among elderly populations.

Given the positive association between RC and disease development, contrasted with the inverse correlation of HDL-C, this study examines the relationship between the RC/HDL-C ratio and incident T2D in Chinese adults, aiming to provide evidence for early prevention and detection to enhance health outcomes.

## 2 Methods

### 2.1 Study population and design

This population-based cohort study utilized electronic health records (EHRs) derived from the Kunshan Elderly Cohort. The cohort collected residents’ health records, annual physical examination data, and follow-up information on chronic diseases via the medical information management system of the regional healthcare consortium. Personal identifiers were anonymized, and data were processed to ensure participant confidentiality. Detailed specifications of the cohort design have been previously documented (23–25). All participants provided written informed consent for the use of their comprehensive EHR data. This study was conducted in accordance with the Declaration of Helsinki, and the study’s protocol was reviewed and approved by the Ethics Committee of the Kunshan First People’s Hospital (Grant No: 2023-03-014-H01-K01).

We enrolled older adult residents who underwent community health screenings in Kunshan between January 2018 and July 2021. Participants meeting any of the following criteria were excluded: (i) insufficient baseline data (RC and HDL-C), (ii) baseline age <60 years, (iii) diagnosis of type 1 diabetes or other specific diabetes types. The final analysis included 7,655 non-diabetic participants.

### 2.2 Exposure and outcome definitions

RC was calculated as total cholesterol (TC) minus HDL-C minus LDL-C (26). The RC/HDL-C ratio was derived by dividing RC by HDL-C (27). The primary endpoint was incident T2D during follow-up, defined by either ICD-10 codes (E11-E14) or fasting plasma glucose (FPG) ≥7.0 mmol/L (24). The time to T2D onset was calculated as the interval between baseline assessment and diabetes diagnosis. For participants without a T2D diagnosis during follow-up, follow-up duration was determined from baseline assessment to the final investigation date.

### 2.3 Covariate definitions

Comprehensive clinical characteristics were extracted from the EHR health screening database, encompassing demographic data, annual lifestyle questionnaires (smoking status, alcohol consumption), and anthropometric measurements (height, weight, waist circumference, blood pressure). Annual laboratory tests for elderly participants included: aspartate aminotransferase (AST), alanine aminotransferase (ALT), blood urea nitrogen (BUN), serum creatinine (Scr), serum uric acid (SUA), TC, TG, and FPG (28). Body mass index (BMI) was calculated as weight in kilograms divided by height in meters squared. According to Chinese BMI classification criteria, weight status categories were defined as: 18.5–23.9 kg/m² (normal weight), 24.0–27.9 kg/m² (overweight), and ≥28 kg/m² (obesity) (29). Estimated glomerular filtration rate (eGFR) was computed using the Chronic Kidney Disease Epidemiology Collaboration equation (30). Hypertension diagnosis criteria included ICD-10 codes (I10-I15), mean systolic blood pressure (SBP) ≥140 mmHg, and/or mean diastolic blood pressure (DBP) ≥90 mmHg (31). Cardiovascular disease (CVD) diagnoses encompassed coronary artery disease (ICD-10: I20-I25) and cerebrovascular disease (ICD-10: I60-I64), documented in the EHR database (28). The chronic disease registry and follow-up database systematically recorded disease incidence, management protocols, and clinical outcomes. Additionally, outpatient prescription data and biochemical measurements improved the accuracy of T2D outcome assessment.

### 2.4 Statistical analyses

Baseline characteristics are summarized as means ± standard deviations (SD) for continuous variables and as counts (n, %) for categorical variables. Participants were grouped based on the presence of T2D or quartiles of the RC/HDL-C ratio. Differences between groups were evaluated using Student’s t-test, analysis of variance, or the chi-square test. Cox proportional hazards models were employed to examine the association between baseline RC/HDL-C levels (as both continuous and categorical variables) and the risk of developing T2D. Variables with clinical or prognostic relevance were included in the models. Model 1 was unadjusted; Model 2 was adjusted for age and sex; and Model 3 further adjusted for BMI, systolic and DBP, FPG, ALT, AST, BUN, Scr, SUA, eGFR, smoking status, alcohol consumption, hypertension, and CVD. The cumulative incidence of T2D events in the RC/HDL-C quartile groups was estimated using the Kaplan-Meier method, and differences between groups were tested with the log-rank test. The dose-response relationship between baseline RC/HDL-C levels and T2D risk was analyzed using restricted cubic splines (RCS) with four knots. Additionally, receiver operating characteristic (ROC) curves were constructed to evaluate the predictive ability of RC/HDL-C and other conventional indicators for incident diabetes. Subgroup analyses were performed stratified by sex (male/female), age (<75/≥75 years), BMI (<24, 24-28, ≥28 kg/m²), CVD (yes/no), hypertension (yes/no), and eGFR (<60, 60-90, ≥90 mL/min/1.73 m²) to assess the stability of the findings. All statistical analyses were conducted using R version 4.2.2 and EmpowerStats software (http://www.empowerstats.com). A two-sided *P* value < 0.05 was considered statistically significant.

## 3 Results

### 3.1 Baseline characteristics

A total of 7,655 participants without T2D at baseline were enrolled, with a mean age of 66.96 ± 4.49 years; among them, 3,689 were males (48.19%) (**Table 1**). Over a median follow-up duration of 3.87 years, 783 participants (10.23%) developed incident T2D.

**Table 1:** Baseline characteristics of the study population.

Characteristics	Total (N=7,655)	Non-diabetes group (N=6,872)	Diabetes group (N=783)	*P* value
Age, years	66.96 ± 4.49	66.84 ± 4.38	68.07 ± 5.25	<0.001
Sex, men	3689 (48.19%)	3358 (48.86%)	331 (42.27%)	<0.001
BMI, kg/m2	24.51 ± 3.23	24.35 ± 3.19	25.87 ± 3.29	<0.001
Smoking				0.161
Never	5751 (75.95%)	5146 (75.74%)	605 (77.76%)	
Former	282 (3.72%)	262 (3.86%)	20 (2.57%)	
Current	1539 (20.32%)	1386 (20.40%)	153 (19.67%)	
Drinking				0.019
None	6032 (79.66%)	5379 (79.17%)	653 (83.93%)	
Light	495 (6.54%)	456 (6.71%)	39 (5.01%)	
Moderate	146 (1.93%)	135 (1.99%)	11 (1.41%)	
Heavy	899 (11.87%)	824 (12.13%)	75 (9.64%)	
SBP, mmHg	139.06 ± 19.19	138.67 ± 19.25	142.50 ± 18.33	<0.001
DBP, mmHg	81.44 ± 10.66	81.35 ± 10.74	82.22 ± 9.97	0.019
ALT, U/L	19.87 ± 12.81	19.44 ± 12.52	23.60 ± 14.56	<0.001
AST, U/L	22.53 ± 10.54	22.31 ± 10.20	24.38 ± 12.98	0.001
BUN, mmol/L	5.67 ± 3.88	5.65 ± 4.06	5.82 ± 1.57	<0.001
Scr, µmol/L	72.57 ± 22.03	72.48 ± 22.43	73.39 ± 18.21	0.146
SUA, mg/dL	324.89 ± 83.97	322.64 ± 83.14	344.66 ± 88.62	<0.001
eGFR, ml/min/1.73 m^2^	84.47 ± 12.98	84.77 ± 12.81	81.80 ± 14.06	<0.001
FPG, mmol/L	5.45 ± 0.66	5.38 ± 0.63	6.06 ± 0.58	<0.001
TC, mg/dL	184.70 ± 35.90	184.86 ± 35.64	183.26 ± 38.09	0.133
TG, mg/dL	145.89 ± 107.39	142.12 ± 102.80	179.04 ± 137.09	<0.001
Hypertension	3776 (49.33%)	3310 (48.17%)	466 (59.51%)	<0.001
Cardiovascular disease	324 (4.23%)	239 (3.48%)	85 (10.86%)	<0.001
RC/HDL-C	0.60 ± 0.66	0.58 ± 0.61	0.79 ± 0.95	<0.001
RC	0.71 ± 0.48	0.69 ± 0.46	0.85 ± 0.59	<0.001

Continuous variables are shown as mean±SD, and categorical variables are presented as n (%) numbers.

RC was calculated as TC minus HDL-C minus LDL-C.

BMI, body mass index; SBP, systolic blood pressure; DBP, diastolic blood pressure; ALT, alanine transaminase; AST, aspartate transaminase; BUN, blood urea nitrogen; Scr, serum creatinine; SUA, serum uric acid; eGFR, estimated glomerular filtration rate; FPG, fasting plasma glucose; TC, total cholesterol; TG, triglyceride; RC/HDL-C, Residual Cholesterol to High-Density Lipoprotein Cholesterol Ratio.

Baseline characteristics stratified by RC/HDL-C quartiles are summarized in **Table 2**: Q1 (0.037–0.292), Q2 (0.294–0.437), Q3 (0.444–0.692), and Q4 (0.692–20.50). Participants in the highest RC/HDL-C quartile exhibited significantly higher BMI, systolic and DBP, ALT, SUA, and FPG levels compared to those in the lowest quartile, with a higher prevalence of hypertension (*P* < 0.001). Conversely, the same group showed lower eGFR levels (*P* < 0.001). Additionally, levels of BUN and alcohol consumption varied significantly across the quartiles of RC/HDL-C (*P* < 0.05). There was no statistically significant association between RC/HDL-C quartiles and smoking prevalence. The highest RC/HDL-C quartile had a higher proportion of females compared to the lowest quartile (*P* < 0.001), while the proportion of males was lower in the highest quartile than in the lowest (*P* < 0.001) (**Table 3**).

**Table 2:** ROC curve of different indicators for predicting the risk of new-onset T2D in the elderly.

Test	AUROC	95% CI	Best threshold	Specificity	Sensitivity
RC/HDL-C	0.601	0.580-0.623	0.455	0.523	0.636
RC	0.596	0.575-0.617	0.600	0.558	0.586
LDL-C	0.542	0.520-0.565	90.875	0.673	0.414
HDL-C	0.427	0.405-0.448	9.668	0.001	1.000
TC	0.484	0.462-0.506	241.688	0.946	0.073

AUROC, area under the receiver operating curve; RC/HDL-C, Residual Cholesterol to High-Density Lipoprotein Cholesterol Ratio; RC, remnant cholesterol; LDL-C, low density lipoprotein cholesterol; HDL-C, high-density lipoprotein cholesterol; TC, total cholesterol.

**Table 3:** Baseline characteristics of the study population according to RC/HDL-C quartiles.

RC/HDL-C	Quartile 1 (0.037-0.292)	Quartile 2 (0.294-0.437)	Quartile 3 (0.444-0.692)	Quartile 4 (0.692-20.50)	*P* value
N	1913	1903	1916	1923	
Age, years	67.03 ± 4.55	67.04 ± 4.57	66.89 ± 4.56	66.90 ± 4.29	0.620
Sex, men	1045 (54.63%)	892 (46.87%)	906 (47.29%)	846 (43.99%)	<0.001
BMI, kg/m2	23.17 ± 3.23	24.23 ± 3.06	24.97 ± 3.13	25.65 ± 2.98	<0.001
Smoking					0.315
Never	1393 (74.06%)	1447 (76.89%)	1461 (76.89%)	1450 (75.96%)	
Former	68 (3.62%)	73 (3.88%)	69 (3.63%)	72 (3.77%)	
Current	420 (22.33%)	362 (19.23%)	370 (19.47%)	387 (20.27%)	
Drinking					0.035
None	1453 (77.25%)	1481 (78.69%)	1543 (81.21%)	1555 (81.46%)	
Light	125 (6.65%)	130 (6.91%)	120 (6.32%)	120 (6.29%)	
Moderate	43 (2.29%)	36 (1.91%)	37 (1.95%)	30 (1.57%)	
Heavy	260 (13.82%)	235 (12.49%)	200 (10.53%)	204 (10.69%)	
SBP, mmHg	136.41 ± 18.85	138.71 ± 18.62	139.57 ± 19.49	141.54 ± 19.46	<0.001
DBP, mmHg	79.88 ± 10.69	81.30 ± 10.54	81.63 ± 10.71	82.93 ± 10.49	<0.001
ALT, U/L	18.40 ± 9.96	19.27 ± 14.66	20.16 ± 13.29	21.63 ± 12.65	<0.001
AST, U/L	22.72 ± 10.26	22.22 ± 11.29	22.50 ± 11.18	22.67 ± 9.32	0.457
BUN, mmol/L	5.90 ± 2.22	5.64 ± 1.45	5.68 + 7.13	5.46 ± 1.45	0.005
Scr, µmol/L	72.98 ± 32.22	71.91 ± 17.02	72.40 ± 17.72	73.00 ± 17.32	0.354
SUA, mg/dl	302.73 ± 79.88	315.97 ± 79.03	330.24 ± 83.29	350.39 ± 85.99	<0.001
eGFR, ml/min/1.73 m2	85.87 ± 13.45	84.55 ± 12.68	84.30 ± 12.53	83.15 ± 13.08	<0.001
FPG, mmol/L	5.39 ± 0.63	5.41 ± 0.65	5.44 ± 0.67	5.55 ± 0.68	<0.001
Hypertension	841 (43.96%)	924 (48.55%)	970 (50.63%)	1041 (54.13%)	<0.001
Cardiovascular disease	68 (3.55%)	89 (4.68%)	83 (4.33%)	84 (4.37%)	0.358
T2D incidence	126 (6.59%)	157 (8.25%)	200 (10.44%)	300 (15.60%)	<0.001

Continuous variables are shown as mean±SD, and categorical variables are presented as n (%) numbers.

BMI, body mass index; SBP, systolic blood pressure; DBP, diastolic blood pressure; ALT, alanine transaminase; AST, aspartate transaminase; BUN, blood urea nitrogen; Scr, serum creatinine; SUA, serum uric acid; eGFR, estimated glomerular filtration rate; FPG, fasting plasma glucose; RC/HDL-C, Residual Cholesterol to High-Density Lipoprotein Cholesterol Ratio.

### 3.2 Relationship between RC/HDL-C and T2D incidence

Cox proportional hazards regression models were used to assess the independent association between RC/HDL-C and incident T2D. RC/HDL-C was identified as a significant risk factor across unadjusted, partially adjusted, and fully adjusted models (all *P* < 0.001). In the fully adjusted model (**Table 4**), each 1-unit increase in RC/HDL-C correlated with a 12% higher risk of T2D (HR = 1.12; 95% CI: 1.06–1.17; *P* < 0.001). When analyzed by quartiles, the multivariable-adjusted HR for incident T2D were 1.37 (95% CI: 1.09–1.73) in Q3 (*P* < 0.05) and 1.78 (95% CI: 1.43–2.22) in Q4 (*P* < 0.001).

**Table 4:** The association between RC/HDL-C and the incidence of T2D during follow-up.

Type 2 diabetes, HR 95%CI, *P* value			
RC/HDL-C	Model 1	Model 2	Model 3
Continuous	1.19 (1.14, 1.24), <0.001	1.20 (1.14, 1.25), <0.001	1.12 (1.06, 1.17), <0.001
RC/HDL-C quartile			
Q1	Reference	Reference	Reference
Q2	1.36 (1.08, 1.72), 0.0098	1.33 (1.05, 1.69), 0.0165	1.20 (0.95, 1.53), 0.1332
Q3	1.77 (1.42, 2.22), <0.001	1.76 (1.41, 2.20), <0.001	1.37 (1.09, 1.73), 0.0073
Q4	2.63 (2.14, 3.24), <0.001	2.58 (2.10, 3.18), <0.001	1.78 (1.43, 2.22), <0.001
P for trend	<0.001	<0.001	<0.001

HR, hazards ratio.

95% CI: 95% confidence interval.

Model 1: no covariates were adjusted.

Model 2: adjusted for age and gender.

Model 3: adjusted for age, gender, smoking status, drinking status, hypertension, cardiovascular disease, BMI, SBP, DBP, FPG, ALT, AST, BUN, Scr, SUA, and eGFR.

**Figure 1:**
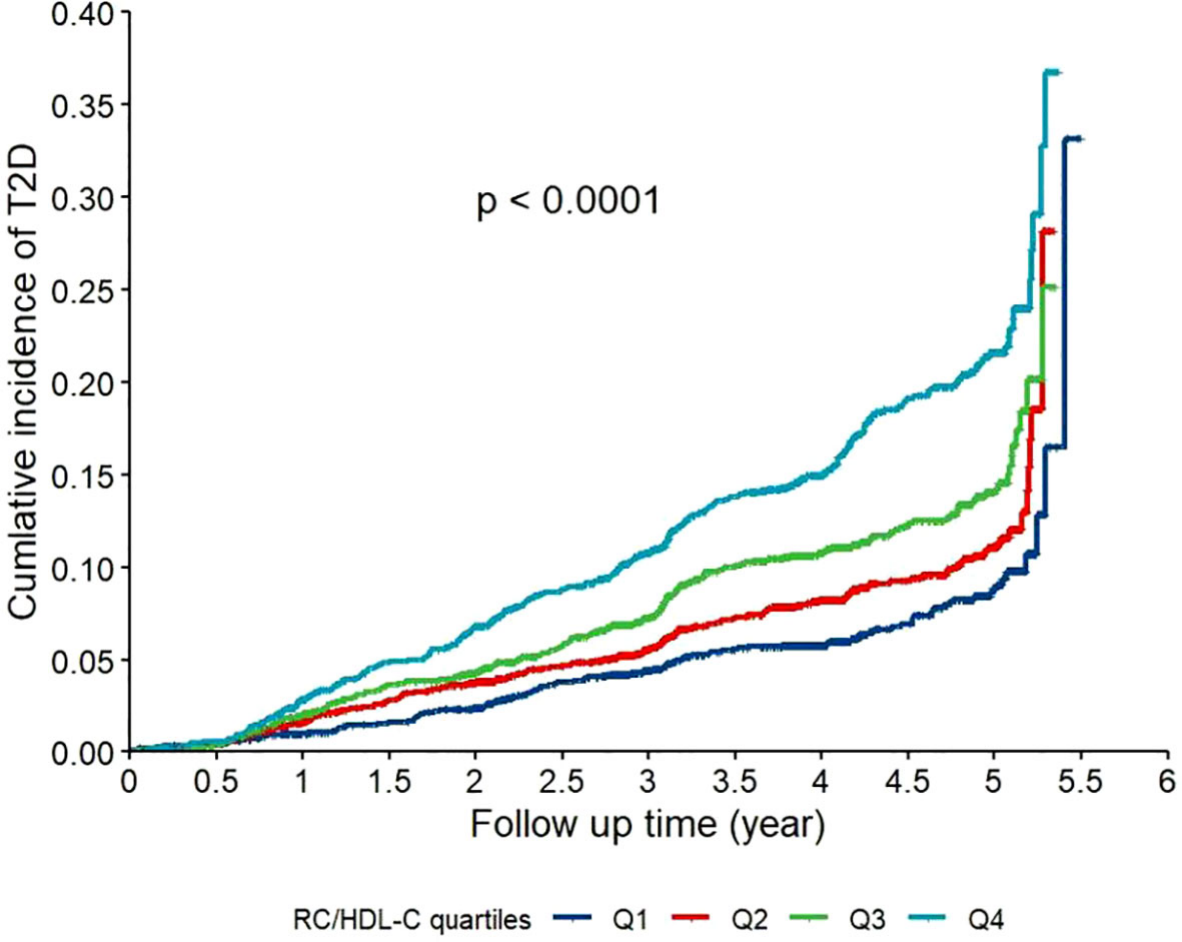
Kaplan-Meier curves for the cumulative incidence of type 2 diabetes (T2D) stratified by quartiles of the residual cholesterol to high-density lipoprotein cholesterol ratio (RC/HDL-C) in the study population (N = 7,655). Participants were categorized into quartiles based on their baseline RC/HDL-C ratio: Q1 (0.037-0.292), Q2 (0.294-0.437), Q3 (0.444–0.692), and Q4 (0.692-20.50). Differences between the curves were assessed using the log-rank test (*P* < 0.001).

**Figure 2:**
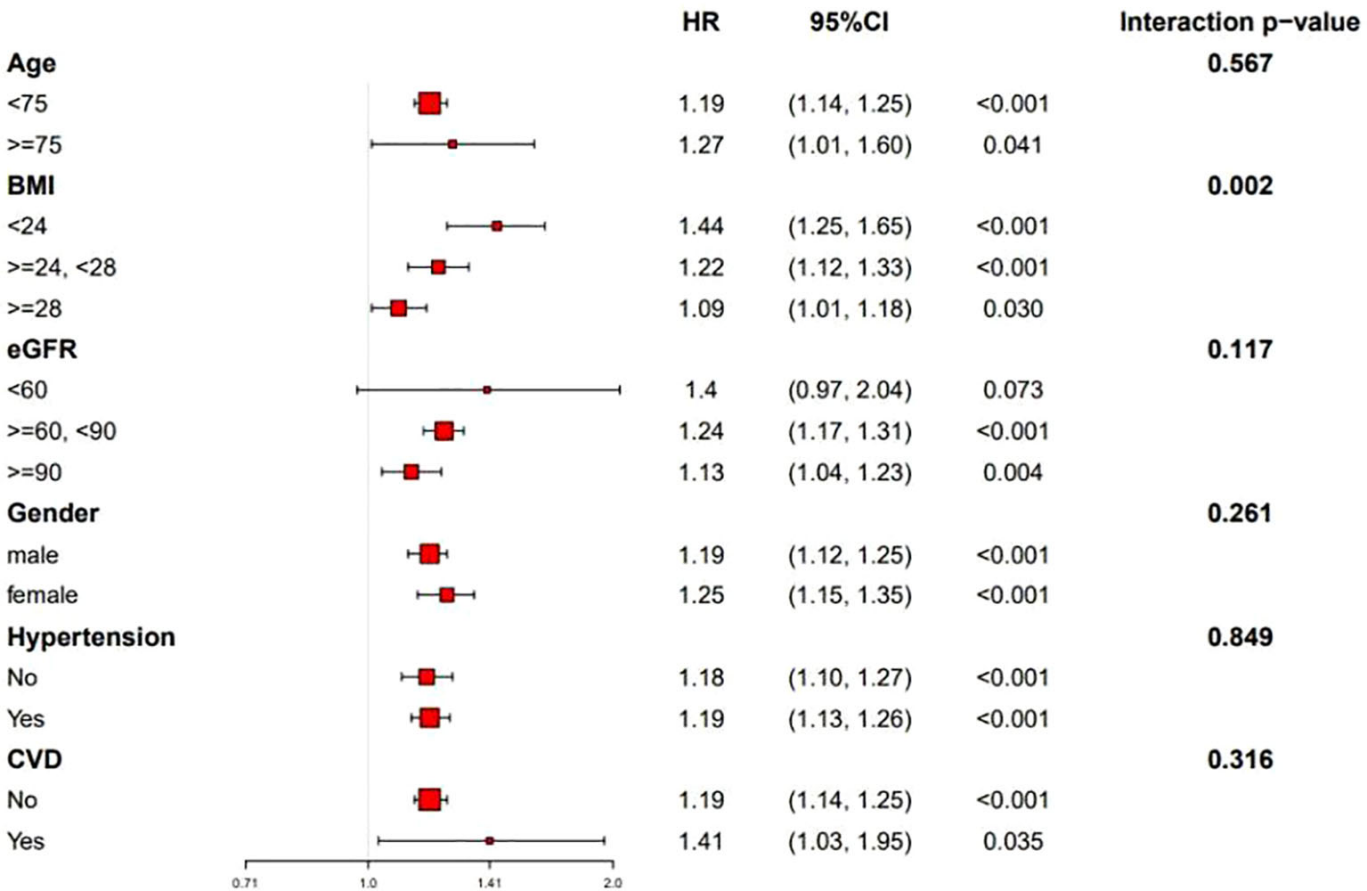
Dose-response relationship between the RC/HDL-C and the risk of incident T2D analyzed using restricted cubic splines (RCS) in a Cox proportional hazards model. The solid line represents the hazard ratio (HR), and the shaded area represents the 95% confidence interval. The reference value (HR = 1) was set at an RC/HDL-C ratio of 0.3. The model was adjusted for age, sex, body mass index (BMI), systolic blood pressure (SBP), diastolic blood pressure (DBP), fasting plasma glucose (FPG), alanine aminotransferase (ALT), aspartate aminotransferase (AST), blood urea nitrogen (BUN), serum creatinine (Scr), serum uric acid (SUA), estimated glomerular filtration rate (eGFR), smoking status, alcohol consumption, hypertension, and cardiovascular disease. The *P* value for the overall association and for non-linearity are presented. *P*-overall<0.05 indicates a significant association between RC/HDL-C and T2D risk. *P*-nonlinear <0.05 indicated a significant nonlinear relationship between RC/HDL-C and T2D risk.

As shown in **Figure 1**, the Kaplan–Meier curves indicated that participants in the highest quartile (Q4) of RC/HDL-C exhibited a significantly greater cumulative incidence of T2D during follow-up (log-rank test, *P* < 0.001). Moreover, as shown in **Figure 2**, RCS analysis revealed a significant non-linear association between the RC/HDL-C ratio and the risk of T2D (*P*_non-linear < 0.001). A positive association was observed between higher RC/HDL-C levels and increased T2D risk up to a threshold of 0.934 (overall *P* < 0.001). Overall, the risk of T2D increased non-linearly with increasing RC/HDL-C levels.

**Figure 3:**
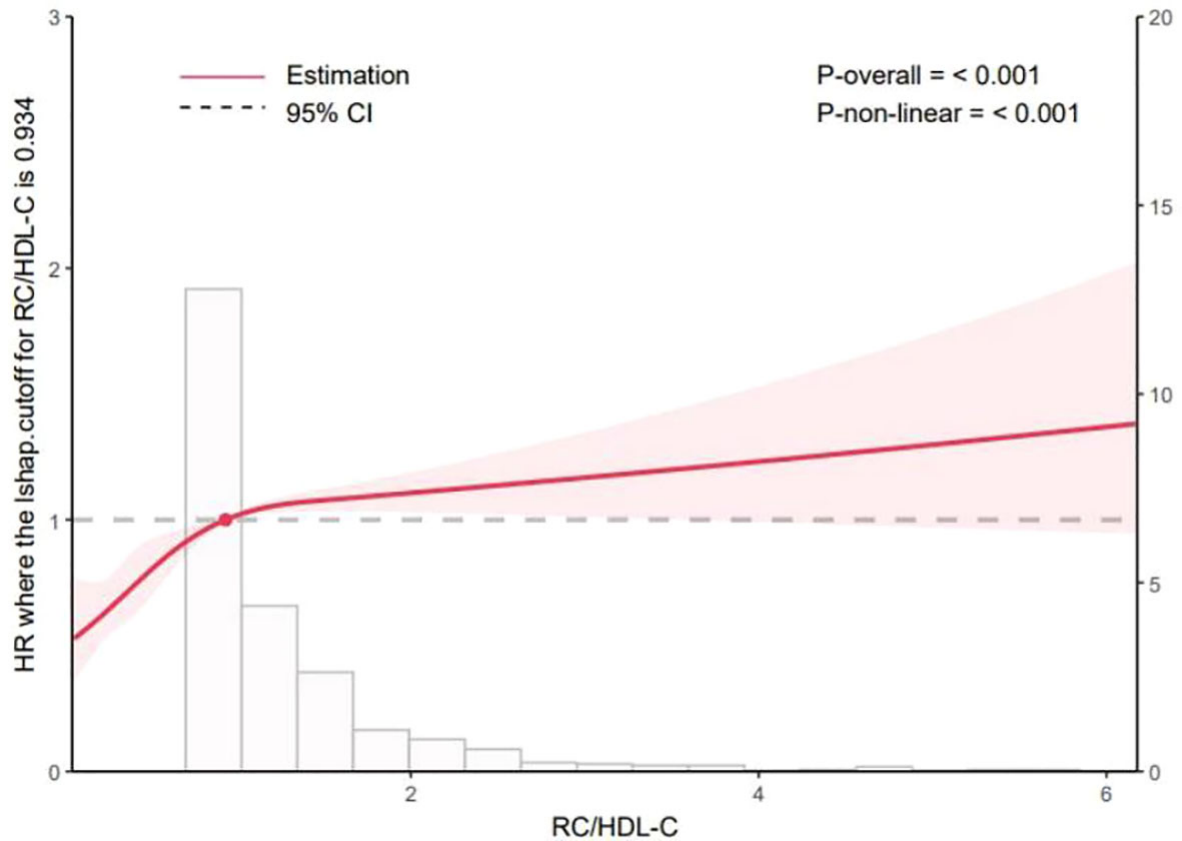
Receiver operating characteristic (ROC) curves comparing the predictive performance of the RC/HDL-C, remnant cholesterol (RC), low-density lipoprotein cholesterol (LDL-C), high-density lipoprotein cholesterol (HDL-C), and total cholesterol (TC) for incident T2D. The area under the ROC curve (AUROC) for each indicator is provided in **Table 2**.

**Table 2** presents the area under the receiver operating characteristic curve (AUROC) for RC/HDL-C and other conventional lipid parameters in predicting incident diabetes among older adults. Compared to RC, TC, HDL-C, and LDL-C, RC/HDL-C demonstrated the highest AUROC (0.601), with a sensitivity of 63.60%, a specificity of 52.28%, and an optimal cutoff value of 0.455 (**Figure 3**), indicating modest but statistically significant predictive ability.

### 3.3 Subgroup analysis

**Figure 4:**
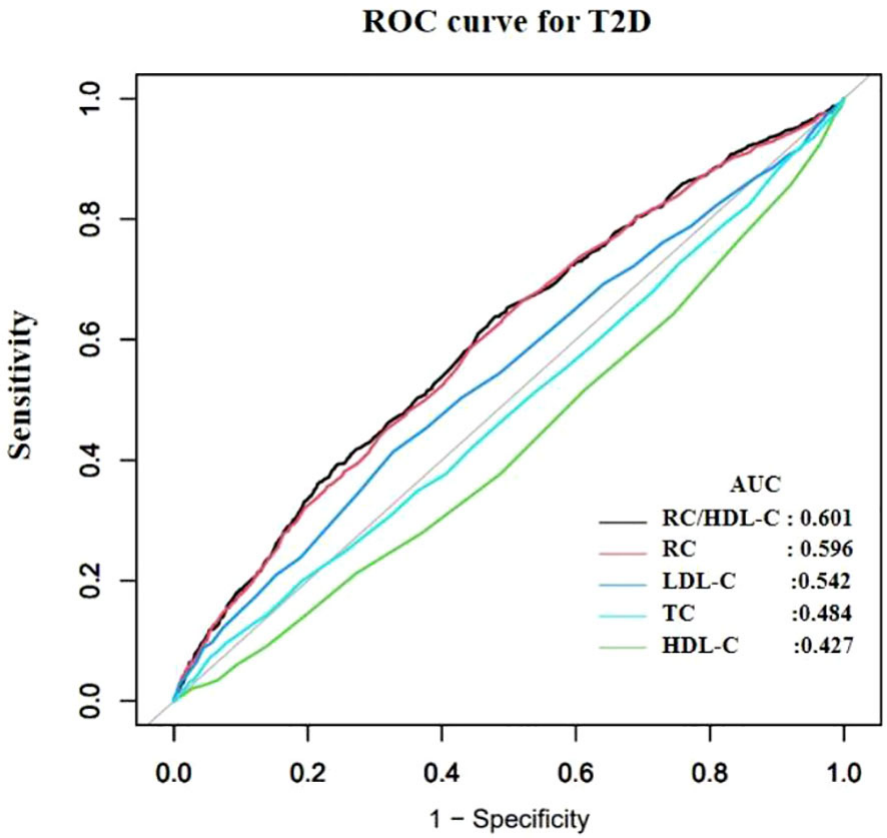
Subgroup analyses of the association between the RC/HDL-C ratio and incident T2D risk (N=7,655). Hazard ratios (squares) with 95% CIs (horizontal lines) are shown for each subgroup. *P* values for interaction are indicated.

Stratified analyses were performed to evaluate the association between RC/HDL-C and T2D risk across subgroups defined by sex (male/female), age (<75 vs. ≥75 years), BMI (<24, 24–28, or ≥28 kg/m²), CVD status (yes/no), hypertension (yes/no), and eGFR (<60/60-90/≥90 ml/min/1.73 m²). As shown in **Figure 4**, the positive association between RC/HDL-C and T2D risk remained consistent across all subgroups, indicating the robustness of the findings.

## 4 Discussion

In this large-scale retrospective cohort study, participants with elevated RC/HDL-C ratios at baseline exhibited a higher incidence of T2D. To our knowledge, this is the first study to examine the association between RC/HDL-C and T2D risk in an older adult cohort. This novel and straightforward biomarker can serve as a non-invasive and easily accessible diagnostic indicator for T2D, and it is expected to supplement established risk factors (such as age and BMI) in comprehensive risk prediction models.

Previous studies have demonstrated that dyslipidemia induces inflammation, endoplasmic reticulum stress, and lipotoxicity, ultimately contributing to IR (32, 33). This relationship may be mediated by excessive cholesterol accumulation in β-cells, leading to dysfunction, impaired glucose tolerance, and reduced insulin secretion. Elevated RC levels have been associated with increased risks of diabetic complications, hypertension, and non-alcoholic fatty liver disease (NAFLD) (34–37). Women exposed to elevated RC levels exhibit increased susceptibility to diabetes, which may be associated with distinct dietary patterns, estrogen status, and cholesterol metabolism. Estrogen deficiency in females promotes atherosclerotic dyslipidemia, visceral adiposity, and IR, collectively elevating the risk of diabetes and cardiometabolic disorders (38, 39). Consistent with these findings, our observations showed that females predominated in the highest RC/HDL-C quartile (*P* < 0.001), whereas males were underrepresented in this quartile relative to lower quartiles (*P* < 0.001). Further mechanistic studies are needed to elucidate the pathophysiological roles of these parameters in the progression of diabetes in the elderly. In summary, the interaction between RC and T2D involves complex mechanisms, including metabolic dysregulation, inflammatory cascades, and vascular pathology.

HDL-C, a multifunctional structural lipoprotein with anti-inflammatory, antioxidant, and cholesterol efflux properties, is widely recognized as “good cholesterol” in cardiovascular pathophysiology (40). HDL-C facilitates reverse cholesterol transport by extracting cholesterol from atherosclerotic vessel walls and delivering it to the liver for metabolic elimination. Reduced HDL-C levels impair glucose homeostasis through mechanisms such as decreased insulin secretion, reduced insulin sensitivity, and downregulation of AMP-activated protein kinase (AMPK) activity (41).

Current longitudinal evidence on the association between the RC/HDL-C ratio and T2D risk remains limited, especially in high-risk elderly populations. Our study identified a positive association between the RC/HDL-C ratio and incident T2D, which remained significant after adjusting for multiple confounders (see Statistical analyses section for details). These confounders included BMI, systolic and DBP, FPG, ALT, AST, BUN, Scr, SUA, eGFR, TC, smoking status, alcohol intake, hypertension, and CVD. Sensitivity analyses confirmed that neither the RC/HDL-C ratio nor T2D risk estimates were substantially affected by these covariates, supporting the robustness of the findings.

The mechanisms underpinning the relationship between the RC/HDL-C ratio and T2D pathogenesis remain poorly elucidated and likely involve multiple pathways. (1) RC transports cholesterol species that are toxic to pancreatic β-cells (PBCs), inducing apoptosis and impairing insulin biosynthesis and secretion. HDL facilitates reverse transport of RC, with elevated HDL levels potentially reflecting both increased clearance and promoting efficient removal of RC — a vital process, given the atherogenic and proinflammatory effects of accumulated RC particles (42). (2) The TG/HDL-C ratio has been established as a robust surrogate marker for IR with strong predictive capacity (43). However, conventional enzymatic assays for TG measure both lipoprotein-bound TG and free glycerol, which limits the ability to directly substitute TG for RC, possibly conferring an analytical advantage to the RC/HDL-C ratio. (3) Sexual dimorphism: Estrogenic activity may dysregulate glucose homeostasis in cerebral and pancreatic tissues, promoting peripheral insulin desensitization (44). Elevated estrogen levels can also drive atherogenic dyslipidemia, visceral adiposity, and IR progression, thereby increasing hepatopathic and cardiometabolic risks (38, 45). These proposed mechanisms require rigorous validation through dedicated experimental studies.

Our findings indicate an independent association between higher RC/HDL-C ratios and increased T2D risk in elderly individuals. This association can be interpreted as a reflection of an imbalance between RC-driven atherogenic/proinflammatory pathways and the protective functions of HDL-C. Specifically, elevated RC may promote IR and β-cell dysfunction through lipid accumulation, inflammatory activation, and oxidative stress, whereas HDL-C supports insulin sensitivity via anti-inflammatory, antioxidant, and cholesterol efflux mechanisms. In our study, restricted cubic spline analysis reveals a nonlinear relationship between RC/HDL-C and T2D risk, with a inflection point at 0.934. This threshold may mark a critical level at which RC–mediated atherogenic and proinflammatory effects accelerate IR and β-cell dysfunction. Values below the threshold suggest that HDL-C can sufficiently counteract the deleterious effects of residual cholesterol through reverse cholesterol transport, whereas values above the threshold imply insufficient counterregulation by HDL-C against the rising RC burden. This pathophysiological framework helps explain the observed nonlinear risk relationship and moderate yet significant predictive value in our elderly cohort. Previous studies reporting nonlinear associations—thresholds for Diabetic retinopathy (DR) and NAFLD at RC/HDL-C of 0.460 and 0.619, respectively—point to threshold effects, beyond which compensatory mechanisms are overwhelmed (46). Prospective cohort studies are required to validate these thresholds for risk stratification and preventive applications, and to assess generalizability across populations. In clinical practice, RC/HDL-C > 0.934 may serve as a practical biomarker to identify high-risk elderly individuals, informing preventive strategies and the frequency of glucose metabolism assessments. For those with RC/HDL-C > 0.934, intensified lifestyle guidance and closer monitoring should be considered, with potential for earlier interventions in high-risk patients. Given our study population of older adults, age-related HDL dysfunction and accumulation of RC may amplify the clinical significance of RC/HDL-C imbalance.

As noted, our study focused on adults aged 60 years and older. The pathophysiological mechanisms linking dyslipidemia to T2D are widely considered to be universal throughout adulthood. Several studies focusing on middle-aged and general adult populations have also reported significant associations between non-traditional lipid markers and T2D (47). This suggests that the biological pathways underpinning our findings are not exclusive to the elderly. However, the RC/HDL-C ratio might hold particular clinical relevance for risk stratification in the elderly due to several age-specific factors: 1)Competing Risks: In the elderly, competing risks of mortality from other conditions (e.g., CVD) can dilute observed associations with T2D. Despite this, we detected a robust independent association, which underscores the potency of this biomarker in this age group. 2)Shift in Pathophysiology: The capacity for βoraci self-replication declines with age, suggesting younger individuals may possess greater regenerative potential (48). Previous studies indicate that younger prediabetic patients are more likely to achieve normoglycemia (49). T2D in the elderly often manifests with more pronounced β-cell function decline rather than pure IR. Future research would be valuable to explore whether RC/HDL-C is more strongly linked to β-cell dysfunction in the context of aging. 3)Polypharmacy and Comorbidities: Our elderly cohort had a high prevalence of hypertension, CVD, and likely concomitant medication use (e.g., statins). Our fully adjusted model demonstrated that the association of RC/HDL-C with T2D was independent of these factors, a finding critical for its potential application in older adults with complex clinical presentations. Conversely, compared to the elderly, younger individuals often adopt unhealthy dietary habits and sedentary lifestyles, which significantly impact IR (50). Future studies directly comparing the predictive value of RC/HDL-C across different age strata are necessary to elucidate potential age-specific effects.

This study’s strengths include its longitudinal assessment of the association between the RC/HDL-C ratio and incident T2D, with significant clinical implications. The investigation was further supported by a median follow-up of 8.2 years, a low attrition rate (<5%), and stable cohort characteristics. Comprehensive sensitivity analyses using multiple models reinforced the robustness of our findings.

Although the RC/HDL-C ratio demonstrated the strongest predictive value among lipid parameters, its AUROC of 0.601 indicates only modest discrimination when used as a standalone biomarker—an expected feature for single biomarkers. The potential advantage lies in its complementarity within established risk-prediction models that integrate multiple factors. This study has not developed or validated an integrated T2D risk prediction model, which represents one of its limitations. The growing role of artificial intelligence (AI) in medicine offers a promising avenue to enhance diabetes screening and risk stratification (51). AI models excel at integrating multifaceted data to improve predictive accuracy, a trend evident across diabetology, cardiology, and musculoskeletal medicine (52–54). In this context, easily measurable and low-cost biomarkers, such as the RC/HDL-C ratio identified in this study, are attractive inputs for scalable screening tools. Future work should explore AI-driven prediction models that integrate RC/HDL-C with additional clinical and anthropometric variables to achieve higher precision in identifying high-risk elderly individuals. Additionally, several other limitations warrant consideration: First, as a retrospective study based on EHRs, key variables such as family history of diabetes, educational attainment, dietary habits, and physical activity levels were not available. This omission may lead to residual confounding, although we adjusted for a comprehensive set of clinically relevant covariates. Future prospective studies that incorporate these important lifestyle and genetic factors are needed to confirm our findings. Second, our study population was exclusively derived from a single center in Eastern China, which may limit the generalizability of our findings to other ethnic or regional groups. Although we identified a significant association between RC/HDL-C and T2D risk, external validation in independent cohorts is necessary to confirm its predictive value before it can be considered for broader clinical application. Future studies should aim to validate our findings in other populations, including international cohorts. Third, the underlying mechanisms linking the RC/HDL-C ratio to diabetic complications require further mechanistic research.

## 5 Conclusion

Elevated RC/HDL-C ratio was significantly associated with a higher risk of incident T2D during longitudinal follow-up in elderly populations. RC/HDL-C shows promise as a research tool for understanding T2D risk, but validation in larger and more diverse populations is needed before consideration of clinical implementation. Future prospective studies should focus on integrating the RC/HDL-C ratio with other established risk factors to develop and validate a robust multivariable diabetes risk prediction model tailored for older adults.

## Data Availability

The dataset involves privacy information of the elderly population in Kunshan and is part of an ongoing follow-up study. To ensure data accuracy and security, and to comply with ethical restrictions, access is restricted. Any requests for access should be submitted to the Ethics Committee of the Kunshan First People’s Hospital. Requests to access these datasets should be directed to Jinting Zhang, 18896660180@163.com.
